# The Development of Hand-Centered Visual Representations in the Primate Brain: A Computer Modeling Study Using Natural Visual Scenes

**DOI:** 10.3389/fncom.2015.00147

**Published:** 2015-12-15

**Authors:** Juan M. Galeazzi, Loredana Minini, Simon M. Stringer

**Affiliations:** Department of Experimental Psychology, Oxford Centre for Theoretical Neuroscience and Artificial Intelligence, University of OxfordOxford, UK

**Keywords:** hand-centered, neural networks, self-organization, reference frames, posterior parietal cortex, area 5d, premotor

## Abstract

Neurons that respond to visual targets in a hand-centered frame of reference have been found within various areas of the primate brain. We investigate how hand-centered visual representations may develop in a neural network model of the primate visual system called VisNet, when the model is trained on images of the hand seen against natural visual scenes. The simulations show how such neurons may develop through a biologically plausible process of unsupervised competitive learning and self-organization. In an advance on our previous work, the visual scenes consisted of multiple targets presented simultaneously with respect to the hand. Three experiments are presented. First, VisNet was trained with computerized images consisting of a realistic image of a hand and a variety of natural objects, presented in different textured backgrounds during training. The network was then tested with just one textured object near the hand in order to verify if the output cells were capable of building hand-centered representations with a single localized receptive field. We explain the underlying principles of the *statistical decoupling* that allows the output cells of the network to develop single localized receptive fields even when the network is trained with multiple objects. In a second simulation we examined how some of the cells with hand-centered receptive fields decreased their shape selectivity and started responding to a localized region of hand-centered space as the number of objects presented in overlapping locations during training increases. Lastly, we explored the same learning principles training the network with natural visual scenes collected by volunteers. These results provide an important step in showing how single, localized, hand-centered receptive fields could emerge under more ecologically realistic visual training conditions.

## 1. Introduction

The brain seems to represent the location of objects in space using a variety of coordinate systems. Consistent with this, several neurophysiological recordings have reported neurons encoding the location of visual targets in different frames of reference. Visual targets are represented initially in a retinocentric or eye-centered frame of reference and in later stages of processing this information is recoded into more abstract, non-retinal coordinate maps that are more suitable to guide our behavior. For example, head-centered, body-centered, hand-centered as well as mixed representations have been reported in different parts of the posterior parietal cortex and adjacent areas (Andersen et al., 1985; Brotchie et al., 1995; Buneo et al., 2002; Pesaran et al., 2006; Bremner and Andersen, 2012).

Similarly, a number of electrophysiological recordings in macaques have also reported neurons with localized and selective responses to stimuli shown in localized regions near the body or parts of the body (i.e., peri-personal space and peri-hand space; Hyvärinen and Poranen, 1974; Rizzolatti et al., 1981, 1988; Graziano and Gross, 1993; Graziano et al., 1994, 1997; Fogassi et al., 1996, 1999; Graziano and Gross, 1998; Graziano, 1999). The visual responding regions of these cells seem to extend from the skin and could be found anchored to different parts of the body (e.g., around the hand, mouth and face). Their response properties do not seem to change with eye movements and the target does not have to necessarily touch the skin to elicit a response.

Cells representing the location of visual targets in hand-centered coordinates have been reported in multiple areas, mostly in the parietal cortex and premotor areas. For planning reach vectors, hand-centered coordinates seem to be the dominant representation in area 5d (Buneo and Andersen, 2006; Bremner and Andersen, 2012). Other hand-centered receptive fields have been found also in ventral premotor areas (Graziano et al., 1997; Graziano, 1999). These cells fire maximally to the location of the target relative to the hand, irrespective of where on the retina this fixed spatial configuration appears. A number of neurophysiological and behavioral studies with human subjects have similarly shown evidence of hand-centered encoding of the location of visual objects near the hands (peri-hand space) in parietal and premotor areas (Makin et al., 2009, 2007; Brozzoli et al., 2011, 2012; Gentile et al., 2011).

Different theoretical approaches have been proposed to reflect the different stages of coordinate transformations and explain some of the response properties found in some neurons of the PPC and premotor areas. A variety of neural network models have been suggested to account for the development of these supra-retinal representations (e.g., head-centered, hand-centered; Zipser and Andersen, 1988; Pouget and Sejnowski, 1997; Blohm et al., 2009; Chang et al., 2009). Some of these models have focused on the development of head-centered responses and despite the computational advantages of these different theoretical efforts, most of this work has been based on supervised learning algorithms, which cannot provide a biologically plausible account of how these properties develop in the cortex. Other computational approaches have suggested a different way of implementing these transformations using neurons behaving like basis function units that could provide an immediate read-out of multiple frames of reference (Pouget and Sejnowski, 1997).

A self-organizing hypothesis to account for how hand-centered representations could occur has been recently proposed (Galeazzi et al., 2013). Here, it was suggested that while the eyes are exploring a visual scene involving a target object in a fixed position with respect to the hand, a form of trace learning would allow the network to associate different views of the same hand-object spatial configuration. This hypothesis was tested using a biologically plausible neural network model, VisNet, of the primate visual system. The architecture of VisNet consisted of a hierarchy of competitive neural layers, with unsupervised learning taking place in the feedforward connections between the layers. These simulation results showed how output cells could learn to respond selectively to the location of targets with respect to the hand, irrespective of where on the retina this spatial configuration was shown.

The simulations presented previously by our laboratory (Galeazzi et al., 2013) involved showing only a hand and single circular object at any one time during training. However, in the real world we rarely encounter one object at the time. In fact, our visual system is mostly confronted with a complex environment consisting of multiple objects. Moreover, in real-world visual scenes the various objects that we encounter throughout our sensory-motor experiences have different shapes and sizes. Nevertheless, cells in the dorsal visual system seem to be able to generalize and form delineated hand-centered visual receptive fields. In this paper we explore whether our model would still be able to develop output cells with single, localized, hand-centered receptive fields when the network is exposed to more realistic images. In the initial simulations presented in Experiments 1 and 2, the training images were comprised of a variety of everyday objects presented simultaneously around a realistic hand. In Experiment 3, we increased the realism further by presenting the hand against a range of completely natural backgrounds during training.

Early research with VisNet (Stringer and Rolls, 2000) has revealed the difficulty for the network to build transform (e.g., position) invariant representations of individual objects when it is trained on cluttered backgrounds. How could the network develop neurons that respond selectively to a single object when it is trained with cluttered images always containing more that one object at a time? Later work has shown that VisNet can in fact form representations of individual objects even when they are never seen in isolation during training (Stringer et al., 2007; Stringer and Rolls, 2008). The statistical decoupling between the different objects works when there is a sufficiently large number of objects and the network is presented with many different combinations of these objects during training. Any particular combination of objects will be seen together only rarely which prevents individual neurons in the output layer from learning to respond to the particular combinations of objects seen during training. Instead, the neurons are forced to learn to respond to the individual objects themselves. The fundamental principle is that competitive learning binds together the features that are seen more often than other less frequent combinations of features in the environment. Thus, the network does not need any prior knowledge of which features belong to a particular object; it self-organizes by learning to respond to the combinations of features that co-occur the most.

We hypothesized that a similar mechanism of statistical decoupling may produce visual neurons that have learned to respond to single object locations in a hand-centered frame of reference. Let us assume that during training the network model is exposed to many images containing the hand with multiple other objects, but where the objects occur in different combinations of hand-centered locations in the different images. Because the objects are always seen with the hand, this forces each of the output neurons to learn to respond to some combination of the hand and hand-centered object locations. However, over many different images there will be a relatively weak statistical link between any two particular hand-centered object locations. These statistics will drive the development of output neurons that have learned to respond to particular spatial configurations of the hand and a single object. That is, these neurons will respond to the presence of an object in only one localized hand-centered receptive field.

To test this learning hypothesis and increase the ecological plausibility of our simulations, three experiments are presented. We first show how the model can develop hand-centered representations using more realistic training images composed of the hand with pairs of objects presented in different hand-centered locations. Many images with different combinations of hand-centered locations were used to ensure adequate statistical decoupling between the different object locations. In a second experiment, we explored whether the output cells of our model developed hand-centered receptive fields that were also somewhat selective to the shape of the object, as well as evaluating how this shape selectivity is affected as the network is trained with more objects. Lastly, in the third experiment we explore whether the network could still develop localized hand-centered receptive fields when the hand is shown against a large collection of different natural background scenes during training. In this case, the background scenes used were entirely natural with no careful control of what objects were present and where they were located.

## 2. Materials and methods

### 2.1. VisNet model

**Figure 1:**
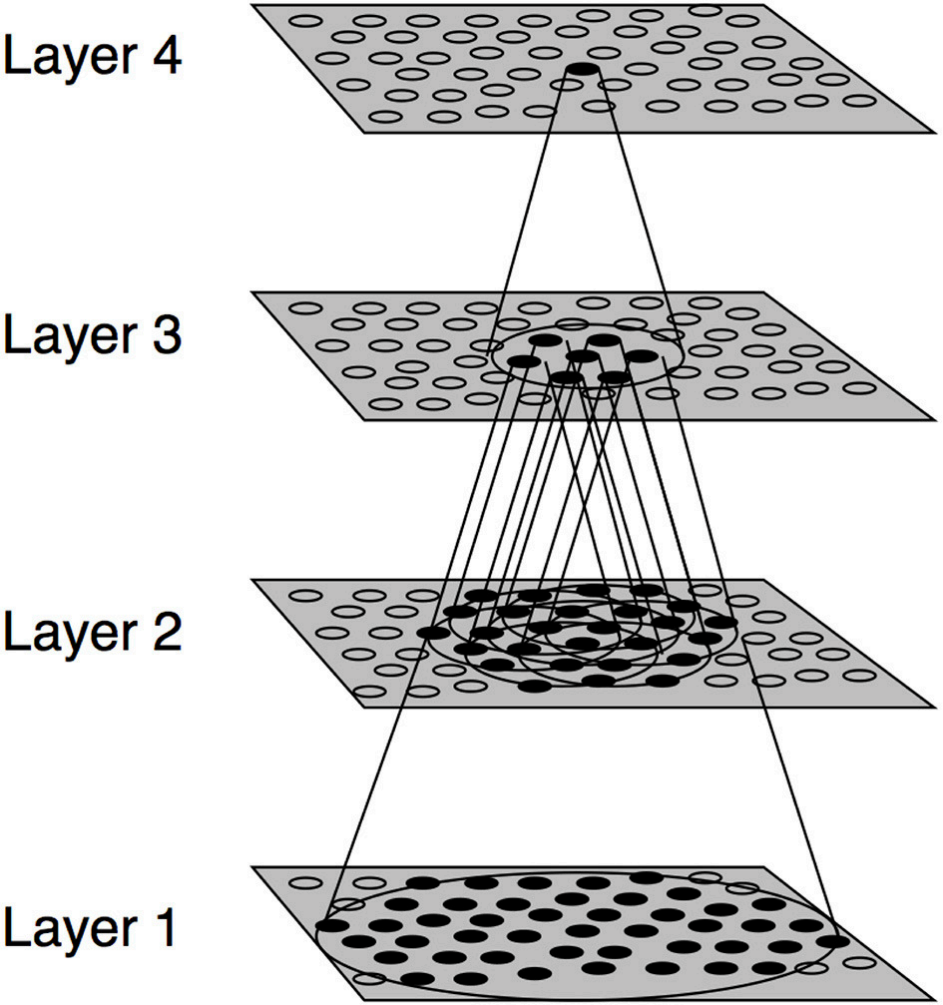
**Stylized image of the VisNet four-layered network**. The architecture of the network shows a hierarchical organization which can be found in the dorsal visual system. Convergence through the network is designed to provide fourth-layer neurons with information from across the entire input retina.

The experiments presented in this paper were conducted using the VisNet model of the primate visual system (Figure 1). VisNet is composed of four feedforward layers of competitive neural networks. Each neuronal layer incorporates lateral competition between neurons which is implemented by local graded inhibition. The synaptic connections between the successive layers of neurons are updated using associative learning. Although VisNet has been often used to model invariance in the ventral visual stream, it has been subsequently applied to simulate visual processes occurring in the dorsal stream (Rolls and Stringer, 2007; Galeazzi et al., 2013; Rolls and Webb, 2014). Both ventral and dorsal streams share architectural similarities, each consisting of a hierarchical series of neuronal layers with competition mediated by inhibitory interneurons within each layer (Rolls and Webb, 2014). The VisNet model is described in the Appendix, more detailed descriptions can be found in Rolls (2008).

In this study the model implements *trace learning*, in which a temporal trace of the previous activity of the neuron is incorporated in the learning rule. This learning mechanism encourages individual neurons to respond to subsets of input images that occur close together in time. We have previously shown how trace learning may allow neurons to develop responses that are selective for the location of visual targets with respect to the hand but invariant to the position of the hand-object configuration on the retina. In particular, we suggested that while the eyes are exploring a visual scene containing a target object in a fixed position with respect to the hand, trace learning would associate together different views (retinal locations) of the same hand-object configuration onto the same subset of output neurons. In this way, different output cells would learn to respond selectively to different positions of the visual objects with respect to the hand, where the neuronal responses were invariant across different retinal locations (Galeazzi et al., 2013).

### 2.2. Information measures

In addition to the response profile of individual neurons, we assessed the network performance using single and multiple cell information theoretic measures. These measures have been used extensively to analyse the performance of the VisNet model in previous work (See Appendix). In this particular case, these measures are used to evaluate whether individual cells in the output layer are able to respond to a specific target location in a hand-centered frame of reference over a number of different retinal locations.

The single cell information metric computes the amount of information conveyed by an individual output layer cell about which of the stimuli has been shown during testing. In this study, a stimulus is defined as one of the different hand-object configurations presented to the network during testing. For example, if an output neuron developed a localized hand-centered receptive field, then it would respond maximally and selectively to the location of an object in a particular position with respect to the hand across all tested retinal locations in which this configuration appears.

On the other hand, the maximal cell information computes the amount of information conveyed by the output population about all of the possible hand-object configurations. This measure verifies whether there is information about all of the testing stimuli across the output layer. For example, if the maximal multiple cell information is reached, this would mean that all the tested hand-object configurations are represented independently by separate output neurons. In other words, the network would develop a variety of hand-centered output cells, each of them with their own localized hand-centered receptive field. These cells would then respond selectively to the location of an object in a particular position with respect to the hand, and all of the tested locations would be represented in the output layer. More details about how these metrics are applied and calculated for this study are provided in Appendix.

### 2.3. Model parameters

For these simulations we used an up-scaled version of the model “retina” (i.e., 256 × 256). Increasing the size of the retina, significantly improves the resolution and therefore the performance of the model. The rest of the parameters are described in Appendix.

## 3. Training and testing procedures

### 3.1. Experiment 1: presentation of the hand with pairings of natural objects

In the first experiment, VisNet was trained on images portraying various spatial configurations of the hand with pairs of natural objects, which were presented against different textured backgrounds. Each of these training images was shifted across different retinal locations during training. We investigated whether these training images could produce output layer neurons with single, localized, hand-centered receptive fields, and which responded invariantly as the neuron's preferred hand-object configuration was shifted across different retinal locations.

#### 3.1.1. Stimuli

**Figure 2:**
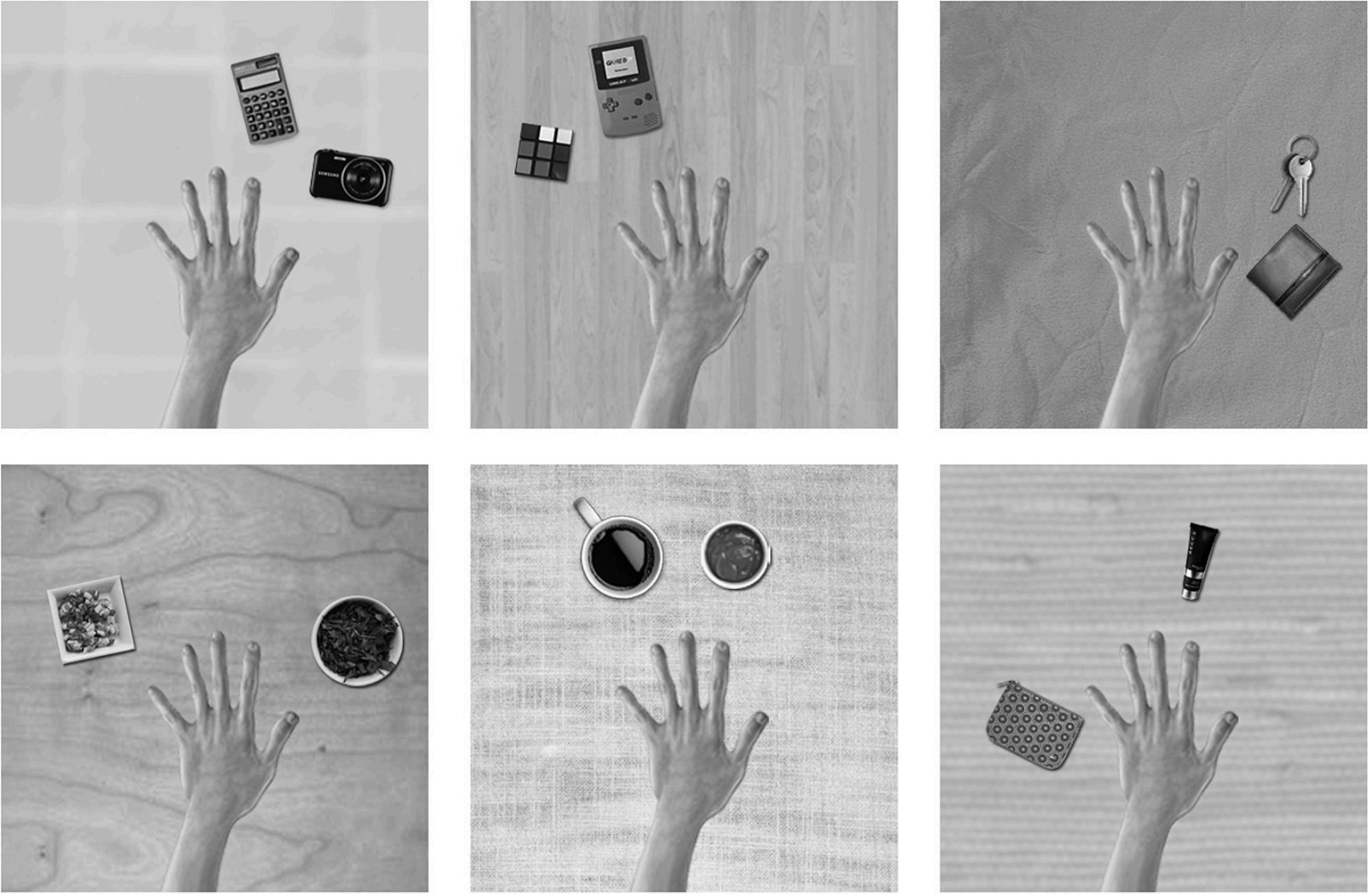
**These are examples of some of the training images used in the first experiment**. A pair of objects would appear in different hand-centered locations simultaneously. The eyes would move exploring the visual scene producing different views of the same configuration across different retinal locations. The six figures represent a sample of the possible images of object pairings generated from the pool of natural objects and textured backgrounds. The relative positions of the hand and the pair of objects are unchanged during the eye movements.

The training images for the first experiment consisted of a hand and two natural objects in different spatial configurations surrounding the hand, all of which were presented against different textured backgrounds. The images of the hand, objects and backgrounds were selected from open source pictures on the internet. The templates were designed, scaled and arranged using Adobe Photoshop software. The images were generated in RGB color and subsequently converted to monochrome using the MATLAB function rgb2gray. Figure 2 shows a sample of some of the training images of hand-object configurations that were generated for this study.

The backgrounds of the images were extended to 512 × 512 pixels for the preprocessing stage. The filtered outputs were then cropped back to the original size 256 × 256. This step is important to avoid possible artifacts or edge effects from the filters in the initial layer of the network.

There was a pool of 42 natural objects to be presented with the hand during training. The centers of all the objects were distributed along a semicircle in six different possible locations around the hand. The images showed all possible pairings of the six hand-centered object locations. The number of possible pairs of object locations may be calculated by

(1) $$\left(\begin{array}{c} n\\ r \end{array}\right)=\frac{n!\;}{\;r!\;(n-r)!}$$

where *n* = 6 and *r* = 2, which gives a total of 15 pairings of object locations. For each such pair of object locations we randomly selected two objects from the pool to be presented in that pair of locations. However, each such pair of objects was presented in both possible arrangements: i.e., object 1 in location 1 and object 2 in location 2, and then object 1 in location 2 and object 2 in location 1. This led to a total of 30 hand-object configurations. Then each of the blocks of these 30 hand-object configurations was presented against one of the 21 different textured backgrounds. This generated a total of 630 images. In order to present the hand-object configurations in different retinal positions, each of these configurations was translated by six pixels at a time across VisNet's retina. During training, the hand was always shown surrounded by a pair of objects and never with a single object in isolation. After training was completed, the images used during testing consisted of the hand and a novel object in a specific position relative to the hand. In the test images, the novel objects were shown in one of the same six hand-centered object locations that were used during training.

#### 3.1.2. Training

The training procedure for this experiment consisted of presenting VisNet with pairs of non-overlapping natural objects displayed around the hand on a textured background. During training, the objects were presented in pairs and never in isolation, (see Figure 2). In order to develop invariant responses across different retinal views, each of the images representing a particular configuration of a hand and objects was trained across five different retinal locations. The image sequences were meant to arise from a series of eye movements and the resulting shifts in the position of the hand and visual objects on the 256 × 256 “retina.” During each of the image sequences, the fixed spatial configuration of the hand and pair of objects was translated six pixels at a time. During the visual exploration of a particular spatial configuration the natural background was always the same. A new background was only used when a new configuration of a hand and objects was presented.

During training, each image was presented to the network in turn. The image was first convolved with the input Gabor filters and the outputs of the Gabor filters are then passed to the first layer of neurons. Next, the firing rates of neurons in the first layer were calculated with soft competition as described in Appendix. Next, the weights of the afferent synaptic connections were updated according to the trace rule given by Equation (A11). This process was then repeated for each subsequent layer of the network. The network was thus trained one layer at the time, starting with layer 1 and finishing in layer 4.

One training epoch consisted of the presentation of all 30 object pairings shown against one of the 21 different textured backgrounds, with each of these images presented across five different retinal locations. Figure 2 shows examples of six training images, each composed of the hand with two natural objects. In these simulations the network was trained for fifty epochs per layer. The learning rates used were 0.1, 0.1, 0.1, and 0.1 in each layer. The number of epochs and learning rates used are the same in all the experiments. For more details on the VisNet parameters, see Appendix.

#### 3.1.3. Testing

**Figure 3:**
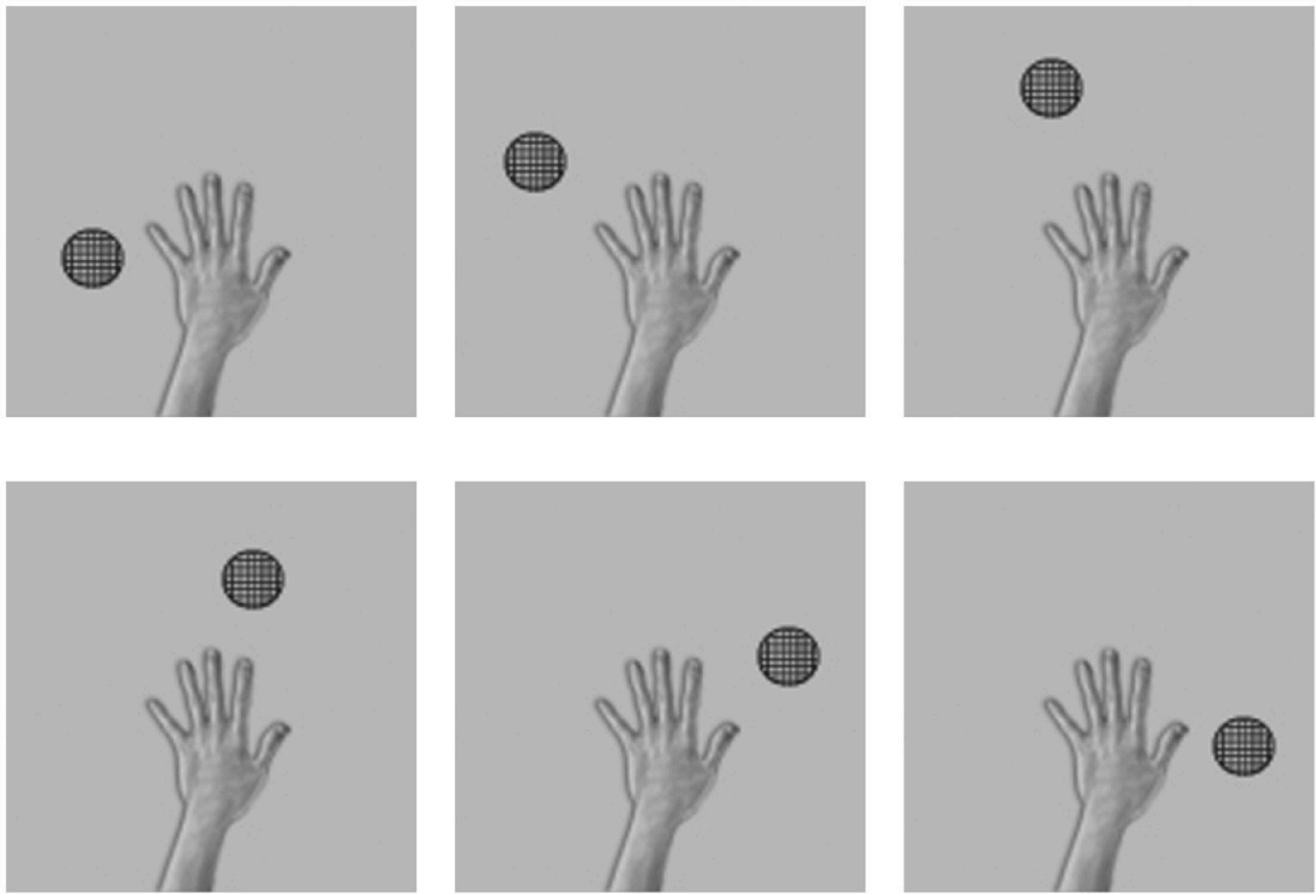
**These are examples of six testing images used in the first experiment to determine the hand-centered receptive fields of output neurons after training**. Unlike the training condition where two objects were presented simultaneously, in this case a single textured stimulus was presented in six different hand-centered locations. The hand-centered object locations were the same as those used during training in the first experiment. Each hand-object configuration would be tested in five different retinal locations.

Throughout the testing phase, the synaptic weights were not changed. Figure 3 shows the six images presented to the network during testing. In order to test whether VisNet has developed translation invariant neurons with a single, localized, hand-centered receptive field, the network was tested with images of the hand and a single circular object presented in only one of the six hand-centered locations at a time. Furthermore, because the goal is to test whether neurons respond to a specific hand-centered location irrespective of the object form, the test images used a simple textured object as shown in Figure 3. During testing, the responses of the output layer neurons were recorded for each of the hand-object configurations shown in Figure 3 presented in each of the five retinal locations.

Lastly, a recent addition to the inspection tools of VisNet enables the user to select an output cell after training and then trace back the connections through layers that have been strengthen by learning. This process can be repeated up until the point that we reach the bank of Gabor filters in the input layer. This permits us to identify which visual features of the input images the selected output cell is responding to most strongly.

### 3.2. Experiment 2: decay of object-selectivity with increased visual training

In the second experiment, we investigated how the shape-selectivity of hand-centered output layer neurons depended on the amount of visual training that the network had received. Specifically, we explored the hypothesis that neurons would become less shape selective as they were trained on larger numbers of objects at their preferred hand-centered location.

#### 3.2.1. Stimuli

**Figure 4:**
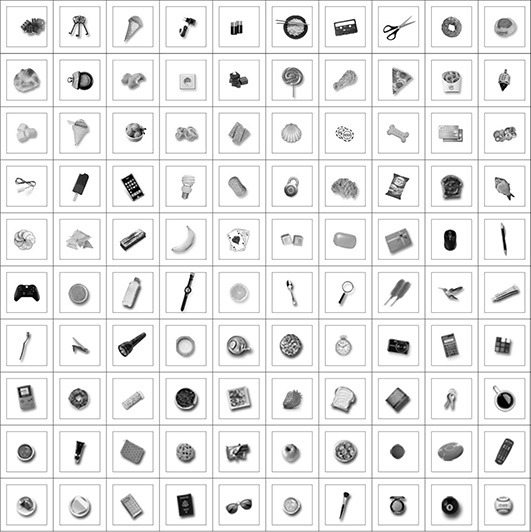
**The figure shows various examples of natural objects that were presented in the same location with respect to the hand during training and testing for the second experiment**. Different objects were used during training and testing. The objects were always presented in the same hand-centered location in order to explore how the shape selectivity of neurons representing that location was affected by the number of objects presented there during training.

The training images for this experiment consisted of the hand presented with a single natural object at a time. The natural object was always presented at the same location with respect to the hand. The objects were drawn randomly from the same pool of 42 natural objects used in the first experiment. The images were generated in RGB color and subsequently converted to monochrome using the MATLAB function rgb2gray. Different simulations were run with increasing numbers (1–8) of natural objects used during training. For each simulation, the network was tested with images of the hand and each of the 100 different novel objects presented in the same hand-centered location on which the network was trained. The objects used during training and testing were not the same. Figure 4 shows examples of the pool of objects used for training and testing. At testing, we recorded the percentage of the 100 test objects that the output neurons responded to. This allowed us to assess the shape selectivity of these neurons.

#### 3.2.2. Training and testing

For this experiment we were interested in exploring whether the output cells that developed visual hand-centered receptive fields could also show shape selectivity, and how this shape selectivity depended on the amount of visual training with different natural objects. We started by training the network with an image of the hand with a single natural object in a particular position with respect to the hand. We then tested the network with a pool of 100 novel objects presented in the same hand-centered location as used during training. Then across further simulations we systematically increased the number of objects that appeared in the same hand-centered location during training. One training epoch consisted of presenting images of the hand with each of the training objects that were used for that particular simulation. After training was completed, the network was tested with the same set of 100 images showing the hand with one of the novel objects. The aim was to investigate how the shape selectivity of neurons that learned to respond to that hand-centered location was affected by the number (1–8) of natural objects seen there during training.

This experiment was not focused on the development of invariant neuronal responses across different retinal locations, and so we trained each image of the hand and object in only a single retinal location. Consequently, we updated the synaptic weights between layers according to the simpler Hebb rule (See Equation A10 in Appendix).

### 3.3. Experiment 3: presentation of the hand against natural backgrounds

In the third experiment, VisNet was trained on images with the hand presented against completely natural backgrounds, which were also shifted across different retinal locations. We investigated whether output layer neurons learned to respond to objects presented in single hand-centered locations, and whether these responses were invariant as the neuron's preferred hand-object configuration was shifted across the retina.

#### 3.3.1. Stimuli

In order to generate our pool of natural visual scenes, we asked four volunteers to provide 10–12 photographs of natural visual scenes from their everyday life in which they would normally use their hands to manipulate objects. All of the volunteers were naive and unaware of the purpose of the study. We provided several examples (e.g., using cutlery in a meal, grasping a cup, etc.) and provided three sample photos in order to give them a general idea of the nature of the scenes we were interested in collecting. We provided further instructions regarding the angle and distance at which the photos should have been taken. The pictures were meant to be taken from a first person point of view and the distance between the objects and the camera had to be at arm's length. Additionally we asked them not to include the image of their own hand in the picture.

**Figure 5:**
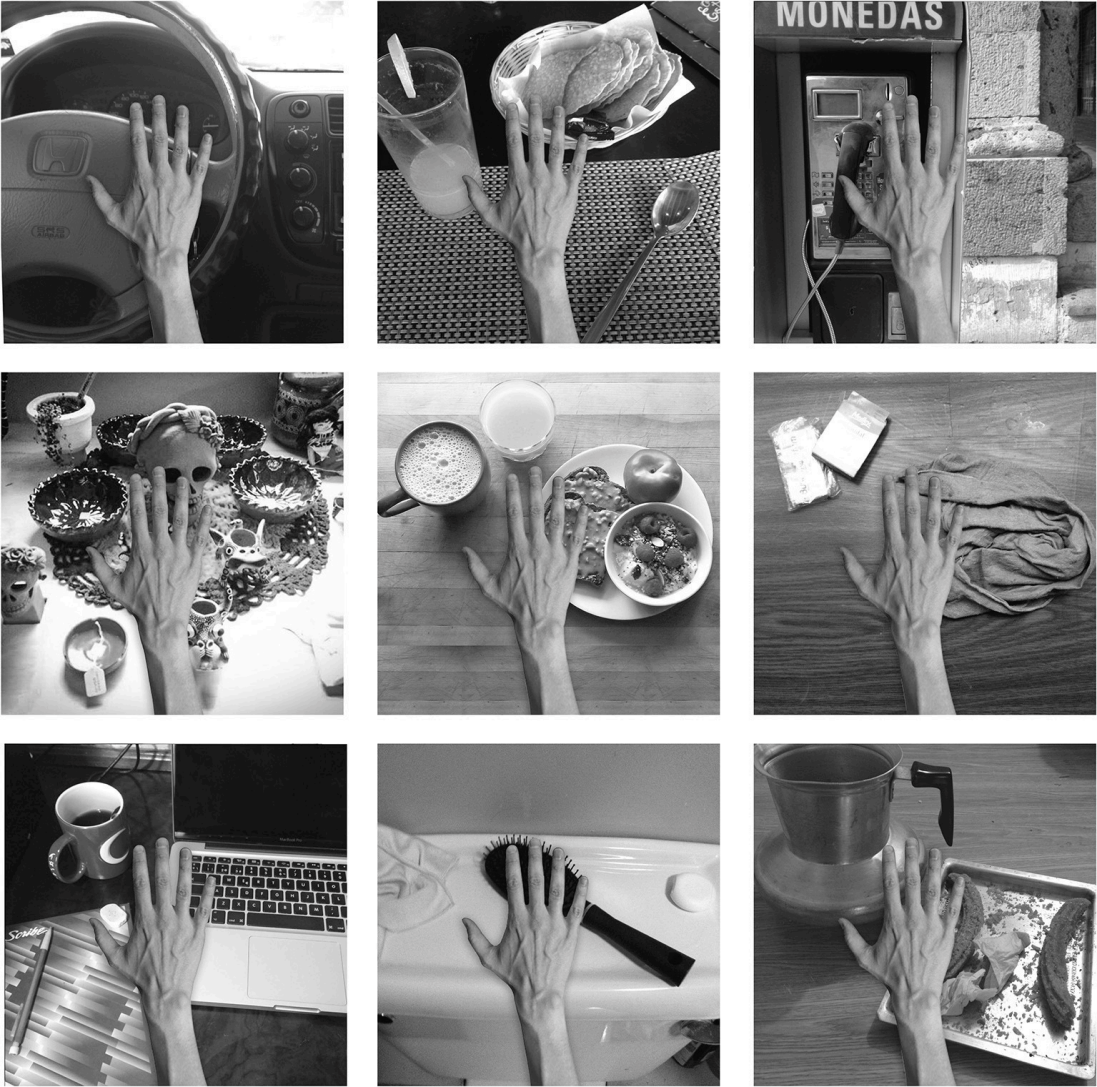
**The figure shows various examples of the hand presented against different natural backgrounds during training in the third experiment**. The position of the hand within each of the backgrounds is unchanged during the eye movements.

The training stimuli for this experiment consisted of images showing a picture of a real hand that was superimposed in all of the natural visual scenes collected by our participants. The templates were scaled and arranged using Adobe Photoshop software. The images were generated in RGB color and subsequently converted to monochrome using the MATLAB function rgb2gray and then resized to a 256 × 256 matrix. Figure 5 shows a sample of some of the training images that were generated. A total of 48 natural images were collected and used for the experiment. In order to present the configurations of the hand and objects in different retinal positions, each of the fixed spatial configurations was translated by five pixels at a time across VisNet's retina within a 3 by 2 grid. That is, for this experiment the sequences included horizontal as well as vertical shifts on the network's retina.

**Figure 6:**
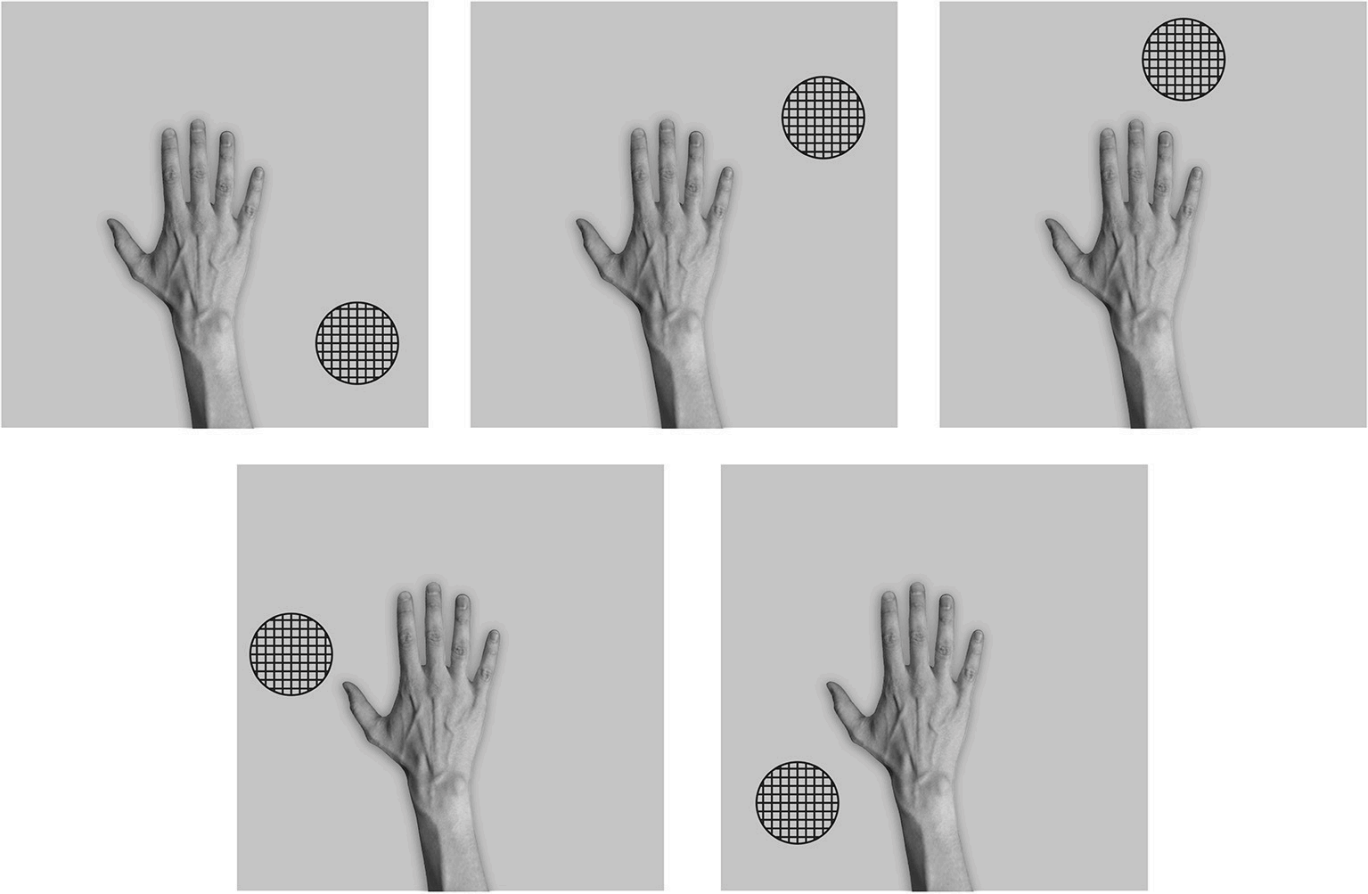
**These are the five test images used after the network had been trained with the hand presented against natural background scenes in the third experiment**. That is, the network was tested with a single textured stimulus presented in five different hand-centered locations. Each hand-object configuration was tested in the six different retinal locations that the hand was originally trained in.

After training was completed, the stimuli used during testing consisted of images showing the hand and a novel textured object in five different positions relative to the hand as shown in Figure 6.

#### 3.3.2. Training and testing

The training procedure for this experiment consisted of presenting VisNet with images of the hand embedded within 48 different natural scenes containing a variety of objects as shown in Figure 5. As in previous simulations, image sequences were meant to arise from a series of eye movements and the resulting shifts in the position of the hand and visual objects on the 256 × 256 “retina.” During each of the image sequences, the fixed spatial configuration of the visual scene is translated both horizontally and vertically by five pixels at a time across a 3 by 2 grid of retinal locations. In the first experiment we shifted the images only horizontally. However, in order to increase the ecological validity of this third experiment, we included a vertical shift of five pixels as well. In this experiment, the synaptic weights were updated according to the trace rule given by Equation (A11). One training epoch consisted of presenting all 48 images in all 6 retinal locations.

Figure 6 shows the images used to test the network after training. In order to test whether VisNet has developed translation invariant neurons with a single, localized, hand-centered receptive field, the network was tested with images consisting of the hand with only a single textured object presented in one of five different hand-centered locations. The responses of the output neurons are recorded with each of these hand-object configurations presented in all six of the retinal locations used during training.

## 4. Results

### 4.1. Experiment 1: presentation of the hand with pairings of natural objects

We studied the responses of the output (fourth) layer cells in VisNet before and after the network was trained on the images of hand-object configurations shown in Figure 2. After the network was trained, the network was tested on the images shown in Figure 3 to determine whether cells in the output layer had developed single, localized hand-centered receptive fields and responded invariantly across the different retinal locations. Information analysis was then conducted on the responses of the cells to all of the test images.

In previous simulations in which VisNet was trained on all possible pairings of objects, it was reported that as the number of objects increased, the statistical decoupling between the objects started to force the network to learn to represent the objects individually (Stringer et al., 2007). However, in the new simulations carried out here the image of the hand was always present with the objects. In this case, the most correlated features would correspond to a combination of features of the hand and features of the trained objects presented in a particular location with respect to the hand. Therefore, individual cells should learn to respond to a particular spatial configuration of the hand and a single hand-centered object location.

**Figure 7:**
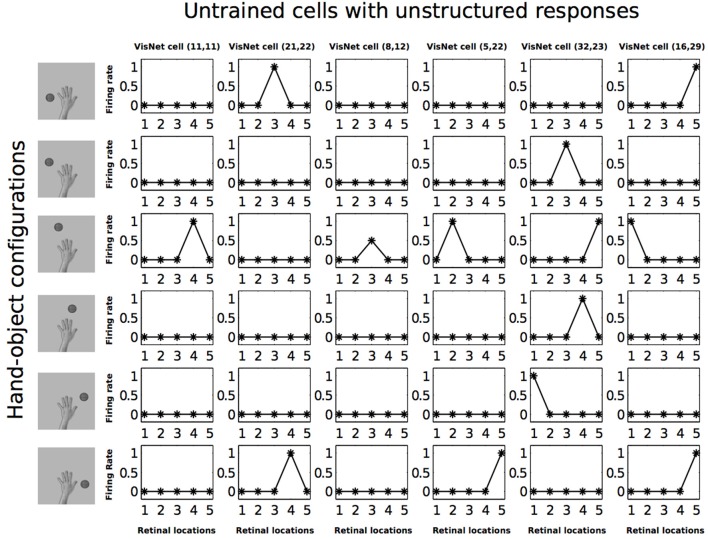
**Firing rate responses from the first experiment before training**. Each of the six columns shows the firing responses of a particular cell. Each row shows the responses of the six cells to one of the six hand-object configurations (shown on the left) over all five different retinal locations shown along the abscissae. It can be seen that each of the six cells initially responds randomly to each of the hand-object configurations over the different retinal locations.

Figure 7 shows the response profiles of six neurons in the output layer of VisNet before training. Following the same conventions of Galeazzi et al. (2013), each of the six columns of plots contains the firing responses of a particular output cell, which are labeled at the top of the column. Whereas the six rows of plots show the responses of the cells to each of the six hand-object configurations presented during testing.

Each plot shows the responses of the given cell to the particular hand-object configuration over the five retinal locations. The x axis in each plot represents the five retinal locations of the hand-object configuration on which the neuron was tested, while the y axis represents the corresponding firing rate of the output neuron. The top row shows the cell responses when a single textured object is presented in the first of the testing locations with respect to the hand. This corresponds to the upper left image in Figure 3. The following rows show the cell responses when the visual object is presented in successive test locations with respect to the hand. The last row corresponds to the configuration displayed in the bottom right image of Figure 3.

**Figure 8:**
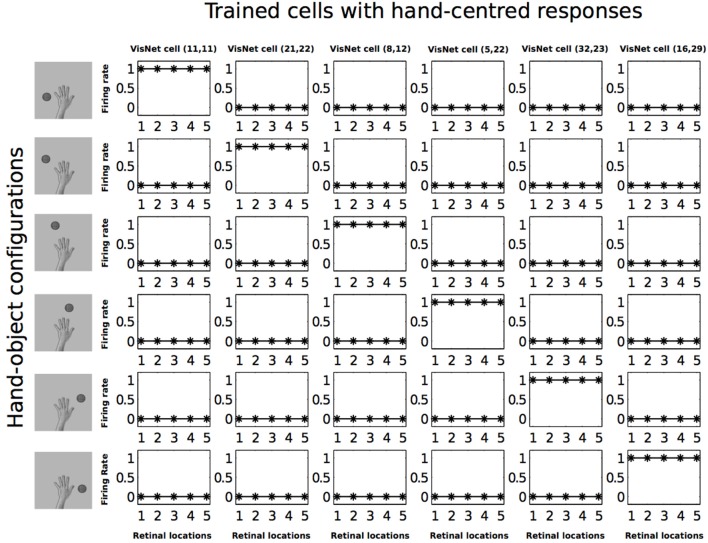
**Firing rate responses from the first experiment after training**. Response profiles of the same six neurons from Figure 7 after training on the images shown in Figure 3. Conventions as in Figure 7. It can be seen that each of the six cells responds selectively to just one of the hand-object configurations, and responds to that configuration over all five retinal positions shown along the abscissae. Moreover, each of the six hand-object configurations is represented by one of the cells.

In Figure 7 we can see that before training, all of the six cells responded rarely and randomly to the different hand-object configurations. The responses do not have a particular ordered structure. In Figure 8 we can see the response profiles of the same six neurons in the output layer of VisNet after training. In this case it can be seen that, after training, each of the six cells has learned to respond to just one of the hand-object configurations, and responds to that configuration over all five tested retinal locations. Furthermore, we can see here already that each of the six hand-object configurations was represented by one of the cells.

**Figure 9:**
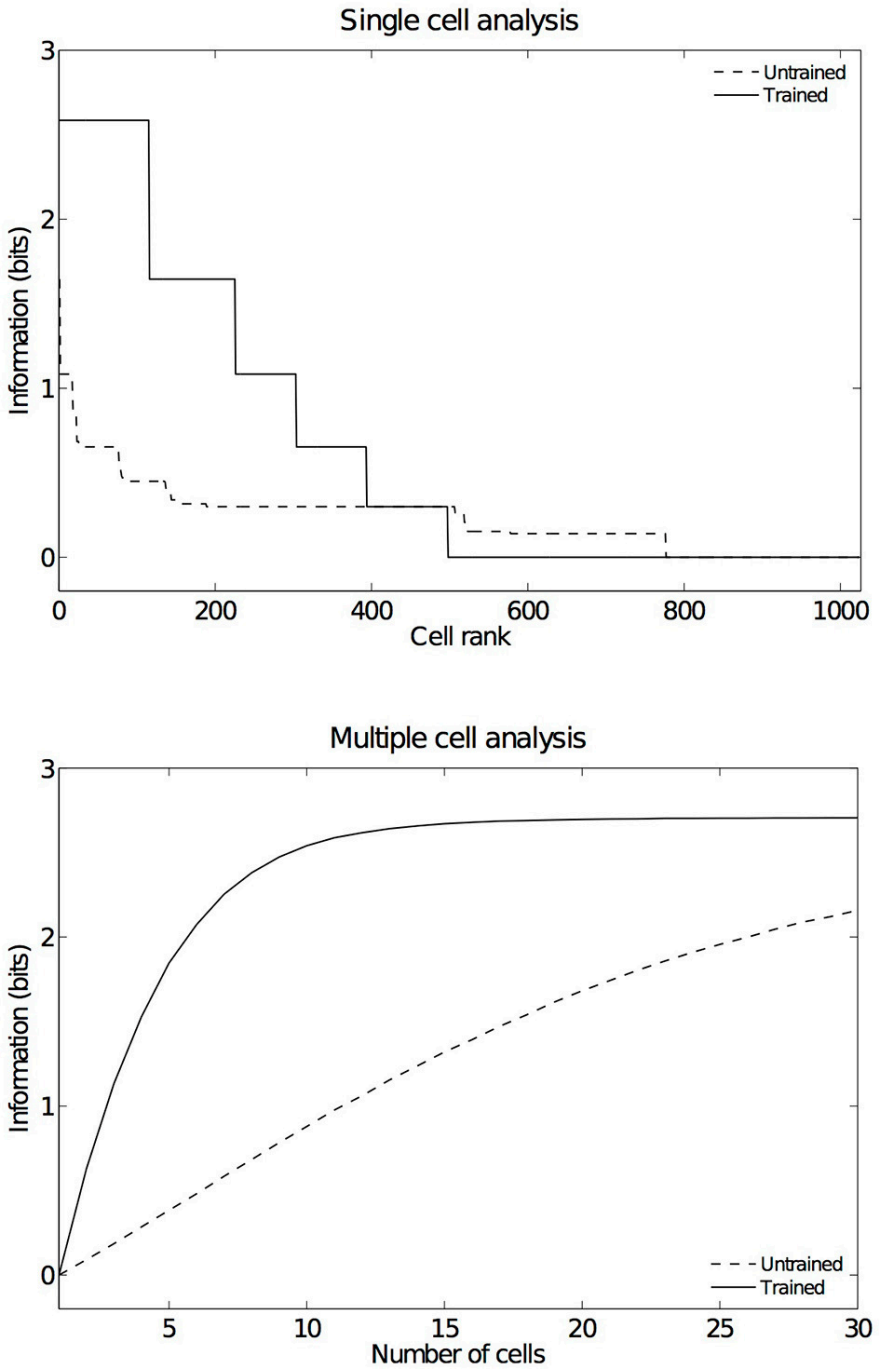
**Information results from the first experiment before and after training**. The upper plot shows the amount of single cell information carried by individual output cells in rank order. After training, it was found that 115 cells reached the maximum amount of single cell information of 2.58 bits. These cells responded perfectly to just one of the six tested hand-object configurations, and responded to that configuration across all five different retinal locations. In the untrained condition no cells reached maximal information. The lower plot shows the multiple cell information measures calculated across 30 cells with maximal single cell information. It can be seen that, after training, the multiple cell information asymptotes to the maximal value of 2.58 bits. This confirms that all six tested hand-object configurations are represented by the output cells.

In order to have an overview of how these configurations are represented across the output cell population, we present the information analysis measures. Figure 9 shows the single and multiple information measures for the output (fourth) layer neurons before and after training with all of the hand-object configurations. The single cell information analysis (Figure 9 top) shows that, after training, 115 neurons conveyed the maximal single cell information of 2.58 bits. These output cells responded to only a single position of the test object with respect to the hand, and responded irrespective of retinal location. The multiple cell information analysis (Figure 9 bottom) shows that, before training, the multiple cell information does not reach the maximal value of 2.58 bits. However, after training we can see that multiple cell information asymptotes to the maximal value, which means that all six of the hand-object configurations are represented by separate cells in the output layer. Figure 8 shows examples of neurons representing each of the six hand-object configurations.

**Figure 10:**
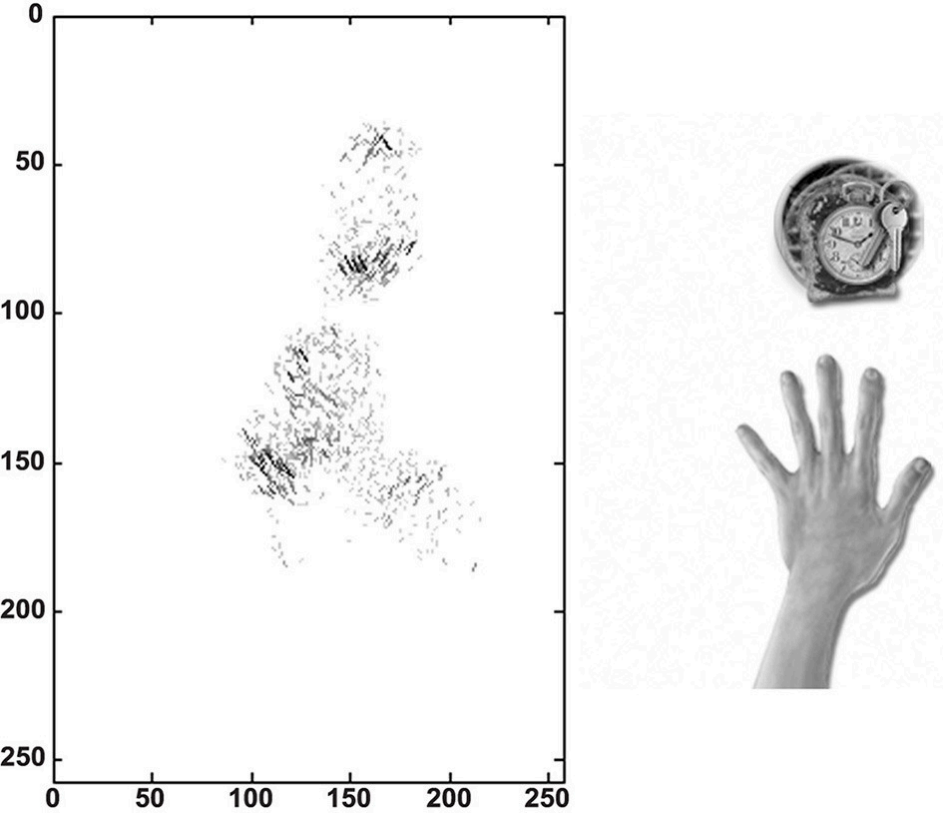
**Tracing back the synaptic connections from a trained output cell to the input Gabor filters in the first experiment**. The left side shows the input Gabor filters that an output cell has learned to respond after training. This is an example of a neuron that represents a hand-object configuration with the object above the hand. In this image the Gabor filters with the strongest connectivity through the layer to the output cell are plotted, where each Gabor filter is weighted by the strengths of the feed-forward connections from that filter through the successive layers to the output neuron. It can be seen that this neuron receives the strongest inputs from a subset of Gabor filters that represent the location of the target on top of the hand. The right side shows the image of the hand and the overlapped images of all the training objects that appeared during training in this hand-centered location.

We traced the strengthened connections from each one of the output cells through successive layers to the input Gabor filters driving that cell. Figure 10 shows the Gabor input filters with strengthened connections to a trained output neuron that had learned to respond to one of the hand-centered locations. On the left side of Figure 10 we can see the Gabor filters that are most strongly driving the responses of the particular output cell. In this example, we show a cell that is representing a subset of Gabor filtered inputs corresponding to the hand, as well as a subset of inputs representing a visual location near the hand. Tracing back the synaptic connectivity in this way enables us to inspect the nature and extension of the hand centered visual receptive field developed by the output cell after training. We can thus determine not only the ability of the cell to represent an individual region with respect to the hand, but also the input features that were extracted from the set of objects shown.

Altogether, the individual cell firing rate responses, the information analysis and the inspection of connectivity in this experiment demonstrate that VisNet is able to develop neurons with a single, localized, hand-centered visual receptive fields even when trained on more realistic images with multiple natural objects shown with the hand against various textured backgrounds. In particular, the principles of statistical decoupling continue to operate successfully under these more ecological training conditions. That is, after extensive training, the output cells learn to respond to the features that are seen more frequently together throughout training. This is a basic property of competitive learning. Since the network is trained on multiple natural objects with the hand against various textured backgrounds, the features that appear more frequently together are the hand (which is always present) and a subset of features that are associated with a particular object location. Consequently, individual output neurons learn to represent a particular configuration of the hand and one object location with separate neurons responding to different hand-centered object locations. However, the statistical decoupling between any two object locations is too weak to allow individual output cells to learn to respond to more than one hand-centered location.

Additionally, the trace learning mechanism enables the network to encode these representations across different retinal locations. Thus, these cells will respond to the same hand-object configuration irrespective of the position of the hand with respect to the body and regardless of the gaze direction. These hand-centered cells will fire maximally as long as the spatial configuration of the hand and an object is the same.

### 4.2. Experiment 2: decay of object-selectivity with increased visual training

In Experiment 1, we were not interested in developing hand-centered cells that were selective to specific objects. On the contrary, we were primarily interested in the development of hand-centered receptive fields where the neuron would respond to the presence of almost any object as long as it was presented within the receptive field. These cells are thought to mostly provide information about the location of an object with respect to the hand, rather than representing the detailed features of the object. However, our simulations do not preclude the possibility that some shape selectivity could arise after training.

In the second experiment, we investigated whether the hand-centered output neurons showed selectivity to the shapes of objects presented with the hand, and how this shape selectivity depended on the amount of training that the network had received with other objects. By testing the network on images with a variety of novel objects in the same hand-centered location used during training, it was possible to assess whether the cells that had learned to respond to that hand-centered location would fire selectively to objects of a particular shape. A number of experiments were performed with sampling different objects during training. The results presented here are taken from one of these experiments and are typical of the effects we observed.

In Experiment 2, eight separate simulations were conducted. Successive simulations used increasing numbers of training objects from 1 to 8, which were always presented at the same location with respect to the hand during training. For each simulation, after training we identified the subpopulation of output neurons that had learned to respond to that hand-centered location. The criterion for classifying a cell as responsive was that its firing rate should reach a threshold of 0.5. Then we tested the network on 100 images of the hand with different novel objects at the same hand-centered location. Each time we recorded whether each of the neurons responded to the new object at that hand-centered location. This procedure was used to reveal how the shape selectivity of the output neurons changed as the network was trained with increasing numbers of objects at their preferred hand-centered location.

**Figure 11:**
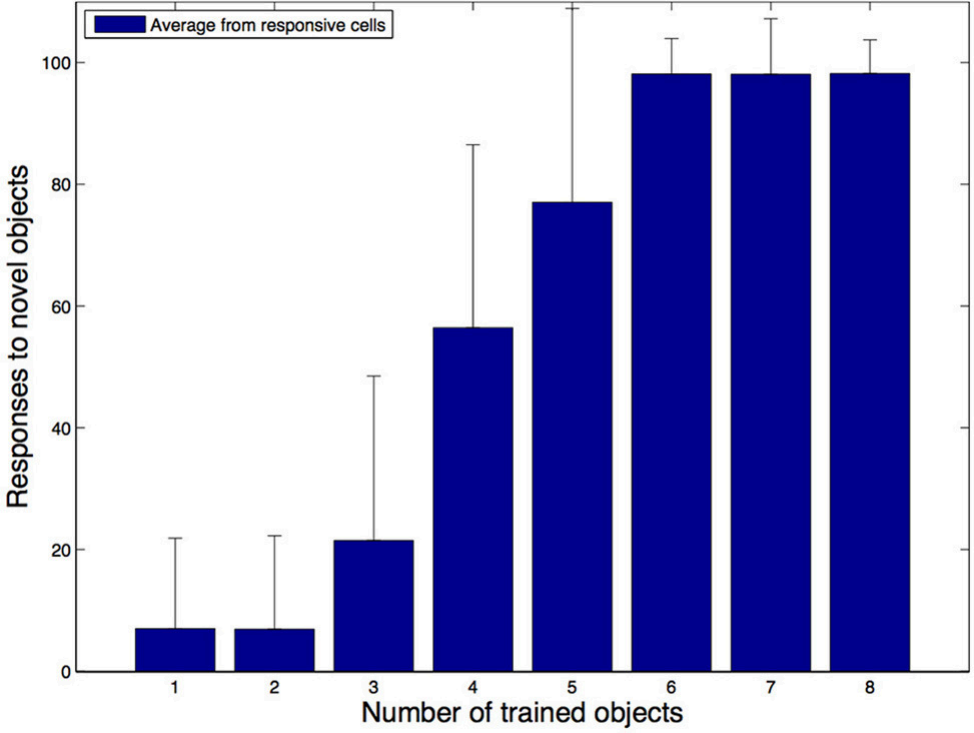
**Simulation results for the second experiment**. In these simulations we explored how the shape selectivity of a subpopulation of hand-centered output neurons is affected as the network is trained with an increasing number of natural objects at their preferred hand-centered location. The plot shows the average number of novel test objects that the subpopulation of output cells respond to as the network is trained with an increasing number of the training objects. It is evident that as the network is exposed to more objects during training, most cells start to lose their shape selectivity and respond to a larger percentage of the novel objects.

Figure 11 shows the average number of novel objects that the hand-centered cells in the network responded to after training as a function of the number of objects that the network has seen at that hand-centered location during training. The ordinate corresponds to the percentage of novel objects that the cells respond to while the abscissa corresponds to the number of objects seen during training. We can see from these simulations that the cells with hand-centered receptive fields started to lose their shape selectivity as they got trained with more and more objects in the same hand-centered location. Even when we still found a few shape selective cells, the proportion of highly selective cells was substantially reduced as the training is increased. This means that most of the cells would respond to the presence of an object in a region of space near the hand regardless of the form of the object.

What learning mechanism leads to a reduction in the shape selectivity of neurons as the network is trained on increasing numbers of objects at the same hand-centered location? When the first object is presented with the hand during training, a small subset of output neurons will win the competition and respond. Then Hebbian associative learning in the feedforward connections within the network will increase the tuning of these cells to respond to that particular object in that hand-centered location. However, when another object is presented in the same hand-centered location, the two objects may share some features in common. The activation of these common features may then cause the same subset of output neurons to respond again because the relevant feedforward connections were strengthened during training with the first object. The effect of this will be to associate the features of the new object with the same output neurons. This process may be repeated with a number of successive different objects presented with the hand. All of the features of these objects will become associated with the same output neurons. Thus, the output neurons gradually lose their selectivity to the form of the objects, and merely respond to any object presented in that hand-centered location. This would produce receptive fields that represent the locations in which the objects appear with respect to the hand, without being particularly selective about the differences between the features of these objects. Thus, as the results show, as the network is trained with more and more objects, the localized hand-centered receptive fields start to lose their shape selectivity and respond to a variety of novel objects as long as they appear within the hand-centered receptive field. This learning process is somewhat similar to continuous transformation (CT) learning (Stringer et al., 2006), which drives the development of invariant neuronal responses by exploiting the similarities between visual stimuli.

Consistent with our results, when we make a comparison at a single-cell neuron level between high-level ventral regions that are shape selective, such as the anterior inferotemporal cortex (AIT) and high level dorsal regions that have been also reported as shape selective (e.g., LIP), it has been found that AIT neurons on average had higher shape selectivity than those of LIP (Lehky and Sereno, 2007). AIT neurons also had significantly more units that were highly selective to shape, whereas LIP had very few neurons that were highly selective to shape.

### 4.3. Experiment 3: presentation of the hand against natural backgrounds

In the third experiment we investigated whether output neurons developed localized hand-centered receptive fields when the network was trained on images containing a hand presented against a natural background scene as shown in Figure 5 and then tested on the images shown in Figure 6.

**Figure 12:**
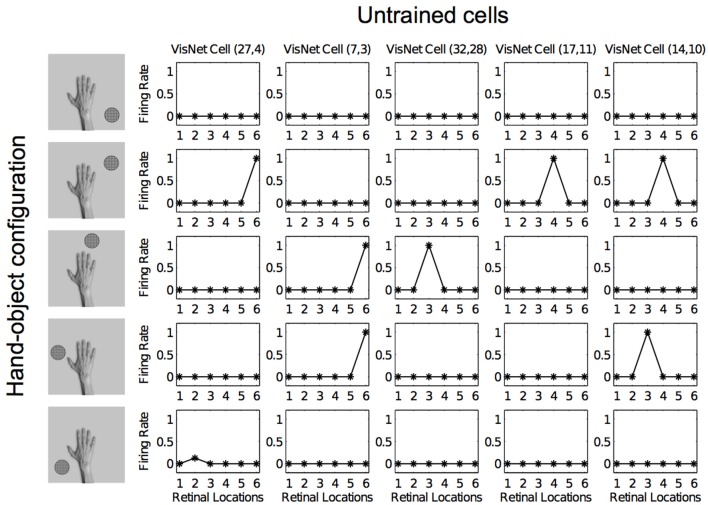
**Firing rate responses from the third experiment before training**. Each of the five columns shows the firing responses of a particular cell. Each row shows the responses of the five cells to one of the five hand-object configurations (shown on the left) over all six different retinal locations shown along the abscissae. It can be seen that each of the five cells initially responds randomly to each of the hand-object configurations over the different retinal locations.

**Figure 13:**
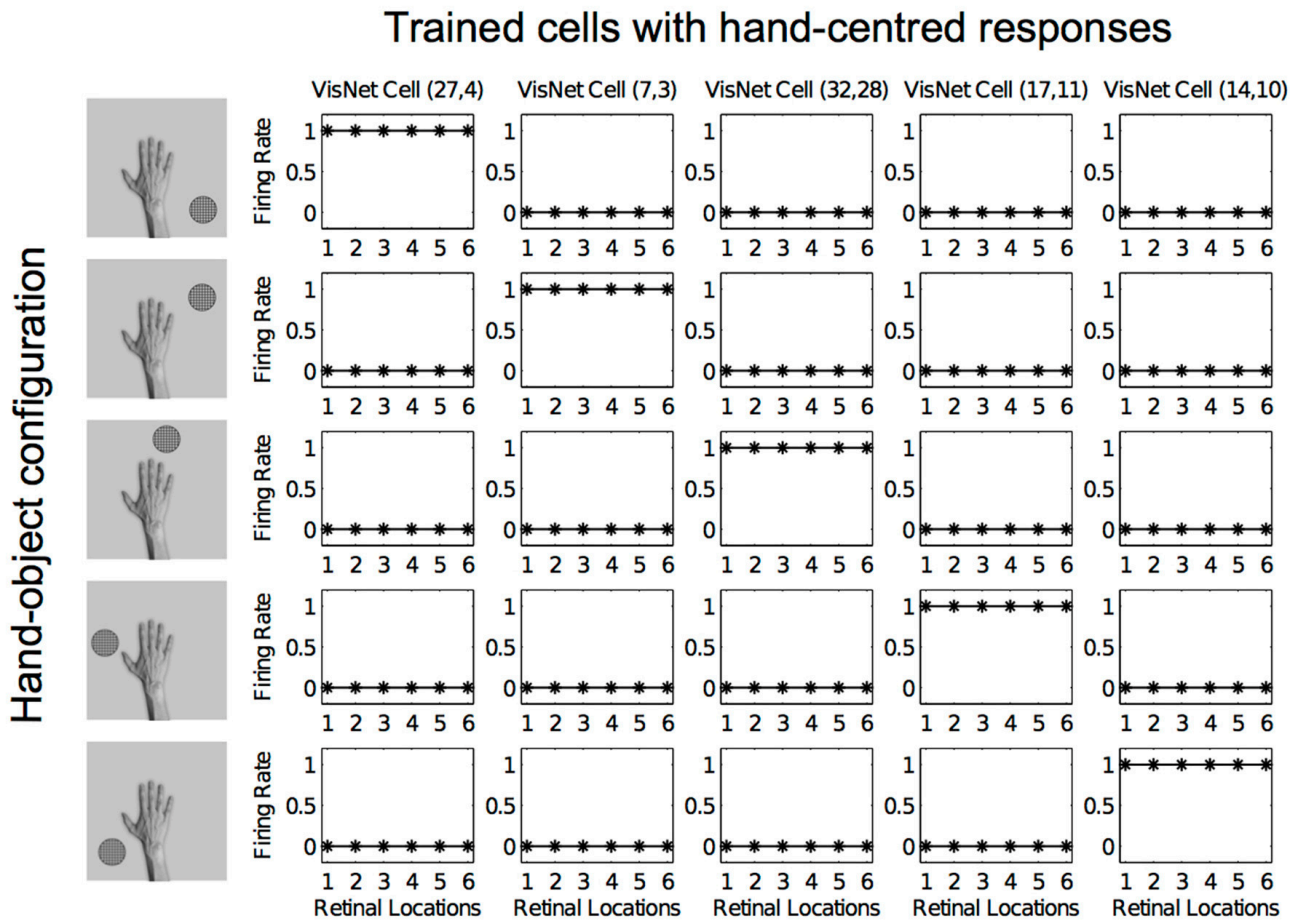
**Firing rate responses from the third experiment after training**. Response profiles of the same five neurons from Figure 12 after training on the images shown in Figure 6. Conventions as in Figure 12. It can be seen that each of the five cells has learned to respond selectively to just one of the hand-object configurations, and responds to that configuration over all six retinal positions shown along the abscissae. Moreover, each of the five hand-object configurations is represented by one of the cells.

Figures 12, 13 show the response profiles of five neurons in the output layer of VisNet before training and after training, respectively. Following the same conventions of the response profiles in Experiment 1, each of the five columns of plots contains the firing responses of a particular output cell, which is labeled at the top of the column. The five rows show the responses of the cells to each of the five hand-object configurations presented during testing. Each plot shows the responses of the given cell to the particular hand-object configuration over six different retinal locations. Before training (Figure 12) none of the cells responded exclusively to any of the hand-object configurations; in fact they responded rarely. However, after training, in Figure 13 we can see that each of the five cells learned to respond exclusively to one specific hand-object configuration, and that these responses were invariant to different retinal locations.

**Figure 14:**
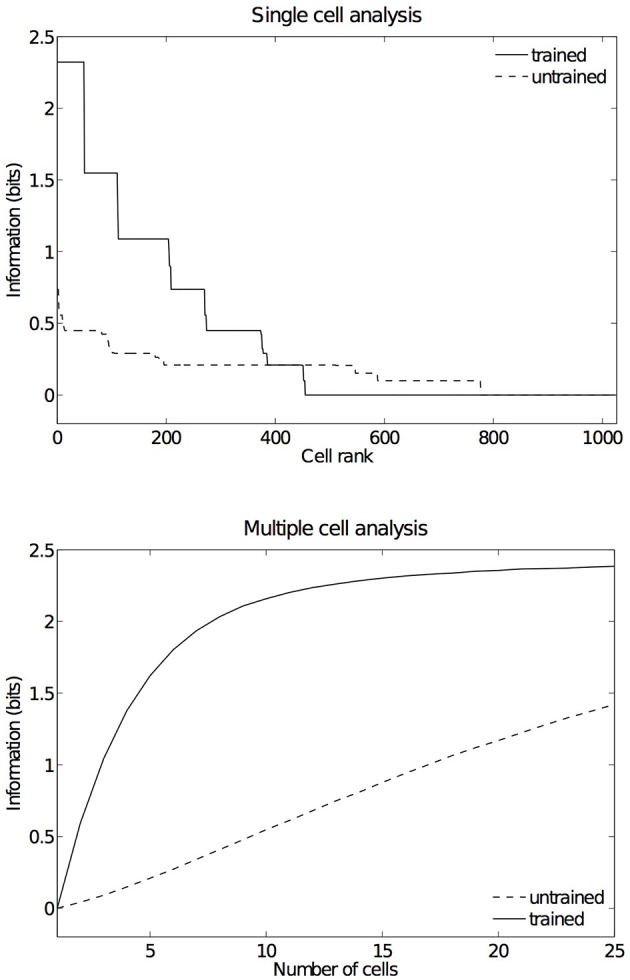
**Information results from the third experiment before and after training**. The information analysis was carried out by testing the network on the five hand-object configurations shown in Figure 6, where each such configuration was presented in six retinal locations. The upper plot shows the amount of single cell information carried by individual output cells in rank order. After training, it was found that 49 cells reached the maximum amount of single cell information of 2.32 bits. These cells responded perfectly to just one of the five tested hand-object configurations, and responded to that configuration across all six different retinal locations. In the untrained condition no cells reached maximal information. The lower plot shows the multiple cell information measures calculated across 25 cells with maximal single cell information. It can be seen that, after training, the multiple cell information asymptotes to the maximal value of 2.32 bits. This confirms that all five tested hand-object configurations are represented by the output cells.

As in the other two experiments presented here, an information analysis was carried out to investigate how these hand-object configurations are represented across the whole population of output cells. Figure 14 shows the single and multiple cell information measures for the output (fourth) layer neurons before and after training the network on images of the hand presented against natural backgrounds. The information analysis was performed by testing the network on the five hand-object configurations shown in Figure 6, where each such configuration was presented in six retinal locations.

Figure 14 (top) shows the single cell information measures for the output layer of neurons. We can see here that, before training none of the cells reached the maximum information. However, after training 49 neurons reached the maximal single cell information of 2.32 bits. This means that these 49 output cells responded selectively to a single localized position of the test object with respect to the hand, and that this response was invariant to retinal location. In Figure 14 (bottom) it is evident that before training the multiple cell information did not reach the maximal value of 2.32 bits. However, after training we can see that the multiple cell information asymptotes to the maximal value, which means that all of the possible hand-object configurations are successfully represented by separate cells in the output layer. In fact, the five cell response profiles after training shown in Figure 13 already confirmed that the network was able to represent each of the five hand-object configurations. The multiple cell analysis simply reaffirms that all five hand-object configurations are represented invariantly across all retinal locations by separate output neurons.

**Figure 15:**
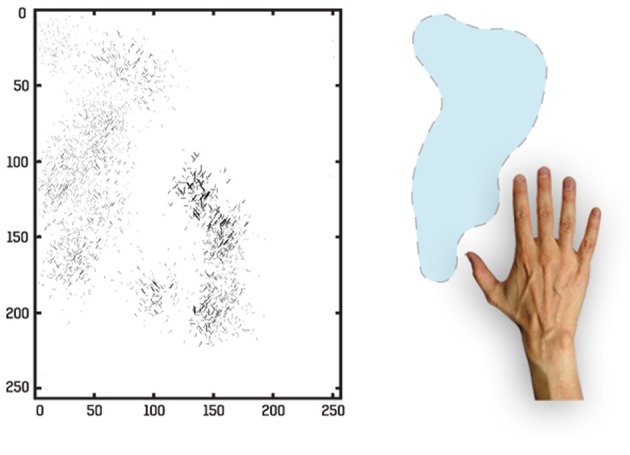
**Tracing back the synaptic connections from a trained output cell to the input Gabor filters in the third experiment after the network was trained on images of the hand presented against natural scenes**. The left side shows the input Gabor filters that an output cell has learned to respond to after training. In this image the Gabor filters with the strongest connectivity through the layers to the output cell are plotted, where each Gabor filter is weighted by the strengths of the feed-forward connections from that filter through the successive layers to the output neuron. The right side shows the image of the hand with the hand-centered receptive field of the neuron shown in blue.

For this simulation we again traced the strengthened connections from each one of the output cells through successive layers to the input Gabor filters driving that cell. In Figure 15 we can see the Gabor input filters with strengthened connections to a trained output neuron that had learned to respond to one of the hand-centered locations. On the left side of Figure 15 we can see the Gabor filters that are most strongly driving the responses of the particular output cell. This cell is representing a subset of Gabor filtered inputs corresponding to the hand, as well as a subset of inputs representing a localized region near the hand. The right side of Figure 15 shows the image of the hand with the hand-centered receptive field of the neuron shown in blue.

## 5. Discussion

In the simulations presented in this paper we have investigated whether VisNet could still self-organize and develop neurons with single, localized hand-centered receptive fields, as the network is trained under more realistic visual training conditions. In these experiments, we have systematically improved the realism of the visual training stimuli in order to test the robustness of the proposed learning mechanism that relies on a combination of statistical decoupling between hand-centered object locations and trace learning in order to drive the development of hand-centered visual representations.

We have shown how some neurons learn to respond to particular spatial configurations of the hand and an object location. Such neurons represent the location of a visual object in the reference frame of the hand. This learning process exploits the statistical decoupling that will exist between different hand-centered object locations across many different images. Furthermore, these neuronal responses can become invariant across different retinal locations by trace learning. This learning rule binds together input patterns which tend to occur close together in time. If the eyes typically saccade around a visual scene faster than the hand moves, then trace learning will bind together the same hand-object configuration across different retinal locations.

In Section 4.1 we began to address how the network might develop neurons with single, localized, hand-centered receptive fields if it is trained on more realistic images containing multiple objects presented simultaneously with the hand. Specifically, we showed that presenting the objects in many different pairs of hand-centered locations during training facilitated the statistical decoupling between different object locations, which in turn forced output neurons to develop localized hand-centered receptive fields. This allowed us to train the network with more than one object presented at a time with the hand.

In Section 4.2 we investigated how the shape selectivity of neurons was affected by the number of objects that the network was trained on at a particular hand-centered location. We proposed that whenever a new object is shown at a particular hand-centered location, then there will likely be some overlap of features with previous objects presented at that location. In such a case, it is likely that some of the same output cells will fire again to the presence of the new object. These cells would get their synaptic weights from the features of the new object strengthened. As the network is trained on more and more objects at the same hand-centered location, this subset of cells gradually learn to respond to most object features at that location and hence lose their shape selectivity. Our simulations suggest the possibility that hand-centered neurons in area 5d and other parts of the posterior parietal cortex may in fact display a range of different degrees of object shape selectivity. The responses of some neurons may be still somewhat selective to shape, while other neurons respond to almost all objects placed within their hand-centered receptive field. Such a heterogeneous population of neurons was in fact observed in our simulations.

Lastly, in Section 4.3 we further increased the realism of the simulations by training VisNet on images of the hand presented against natural visual scenes. Unlike the previous simulations where the hand-centered object locations were carefully controlled, this time the objects could appear in any location around the hand. Furthermore, there was also more variability in the relative size of the objects and their distance to the hand. Given the richness of the visual training scenes in Experiment 3, the output cells showed more spatial heterogeneity in their receptive fields. For example, as shown in Figure 15, one of the particularly interesting differences in this simulation result was that the localized receptive fields near the hand had irregular and idiosyncratic shapes, some of them covering larger areas surrounding the hand.

Altogether, the results from the experiments presented here showed how individual output cells could develop single, localized, hand-centered visual receptive fields which are invariant to retinal location. This occurred even when the network was trained on more realistic visual scenes with multiple objects presented simultaneously with the hand, or even with the hand presented against complex natural backgrounds. This is an important step to show how these hand-centered representations could emerge from the natural statistics of our visual experiences and under more realistic training conditions. More importantly, we showed that this can be achieved using an unsupervised learning mechanism where the synaptic weights are updated in a biologically plausible manner using locally available information such as the pre- and post-synaptic neuronal activities.

### 5.1. Future directions

In the simulations described in this paper, the hand was always presented to the network in the same pose. In future work, we plan to run simulations in which the hand is seen in different postures. For example, the network might be trained on sequences of images as the hand rotates to pick up a series of objects. In this case, we hypothesize that neurons may develop a diverse range of response properties. Some neurons may become selectively tuned to the presence of a visual target with respect to just one pose of the hand, while other neurons could develop pose invariant responses through an invariance learning mechanism such as trace learning (Földiák, 1991; Rolls, 1992) or continuous transformation learning (Stringer et al., 2006).

In this paper we were primarily interested in the visual development of such hand-centered representations using a self-organizing approach. Therefore, the input provided to the network about the location of the hand and target was presented visually. However, in the brain the positional information of the location of the hand is integrated using inputs from different modalities, including tactile and proprioceptive signals. In this study we did not explore the role of these different incoming signals. Nevertheless, we hypothesize that they could in some cases facilitate the statistical decoupling and formation of localized hand-centered receptive fields. For example, tactile feedback from the touch of an object will be generally congruent with visual signals representing the hand-centered location of the visual object. In future work, we plan to integrate signals from other modalities such as tactile and proprioceptive information to explore their role in the development of hand-centered representations.

As we mentioned in the Introduction, a variety of regions have been reported as encoding target positions in a hand-centered frame of reference. However, there might be functional differences between these different hand-centered representations (De Vignemont and Iannetti, 2015). It is, for example, unclear how the hand-centered encoding of reach vectors reported in area 5d by Bremner and Andersen (2012) may relate or differ from other hand-centered and peri-hand representations reported in different regions (Graziano et al., 1994, 1997; Graziano and Gross, 1998; Graziano, 1999). The intention to reach to a desired location might be crucial for the hand-centered cells in area 5d, while the mere presence of an object near the hand could be sufficient to elicit a response from a hand-centered cell in PMv even if there is no intention to interact with it. Some of the behavioral tasks and data analysis from these different studies are not immediately comparable and involve a limited set of experimental conditions. This makes it difficult to disentangle not only the frame of reference in which a particular cell encodes the location of a target, but also how visual, proprioceptive, tactile and motor signals are weighted and integrated during the task. Furthermore, many of these cells may very well have interesting dynamical properties in which the frame of reference could be varying during different moments of the task (Bremner and Andersen, 2014).

## Funding

This work was funded by The Oxford Foundation for Theoretical Neuroscience and Artificial Intelligence and the Consejo Nacional de Ciencia y Tecnología (CONACYT, Scholar:214673 grant no. 309944, http://www.conacyt.gob.mx).

### Conflict of interest statement

The authors declare that the research was conducted in the absence of any commercial or financial relationships that could be construed as a potential conflict of interest.

## Appendix

### A. VisNet architecture and parameters

#### A.1. VisNet

The VisNet model consists of a hierarchical series of four feedforward layers of competitive networks. Within each neuronal layer there is lateral competition between neurons implemented by local graded inhibition. During training, there is associative learning at the synaptic connections between the successive layers of neurons (See Figure 1). In VisNet, natural visual images are first passed through an array of filters mimicking the response properties of V1 simple cells, and subsequently these images are fed to the first layer of the network architecture. The forward connections to individual cells are derived from a topologically corresponding region of the preceding layer, using a Gaussian distribution of connection probabilities. These distributions are defined by a radius which will contain approximately 67% of the connections from the preceding layer. This leads to an increase in the receptive field size of neurons through successive layers of the network hierarchy. The network dimensions used for this study are shown in Table A1. The architecture captures the hierarchical organization of competitive neuronal layers that is common in both the dorsal and ventral visual systems.

**Table A1:** **Network dimensions showing the number of connections per neuron and the radius in the preceding layer from which 67% are received**.

	**Dimensions**	**Number of connections**	**Radius**
Layer 4	32 × 32	100	12
Layer 3	32 × 32	100	9
Layer 2	32 × 32	100	12
Layer 1	64 × 64	100	12
Retina	256 × 256 × 16	–	–

The simulations were conducted utilizing an updated version of the VisNet model (Rolls and Milward, 2000; Rolls, 2008). Before presenting the stimuli to VisNet's input layer, they are pre-processed by an initial layer representing V1 with a dimension of 256 × 256 where each *x, y*-location contains a bank of Gabor filter outputs *g* corresponding to a hypercolumn generated by

(A1) $$g\left(x,y;\lambda ,\theta ,\psi ,\sigma ,\gamma \right)=\exp\left(-\frac{x\prime^{2}+\gamma^{2}y\prime^{2}}{2\sigma^{2}}\right)\cos\left(2\pi \frac{x\prime }{\lambda }+\psi \right)$$

(A2) x′=xcosθ+ysinθ

(A3) y′=−xsinθ+ycosθ

for all combinations of λ = 2, γ = 0.5, σ = 0.56λ, θ∈{0, π∕4, π∕2, 3π∕4} and ψ∈{0, π, −π∕2, π∕2}.

The activation *h*_*i*_ of each neuron *i* in the network is set equal to a linear sum of the inputs *y*_*j*_ from afferent neurons *j* weighted by the synaptic weights *w*_*ij*_. That is,

(A4) $$h_{i}=\sum_{j}w_{ij}y_{j}$$

where *y*_*j*_ is the firing rate of the presynaptic neuron *j* in the preceding layer, and *w*_*ij*_ is the strength of the synapse from neuron *j* to neuron *i*.

Within each layer competition is graded rather than winner-take-all, and is implemented in two stages. First, to implement lateral inhibition the activation of neurons within a layer are convolved with a spatial filter, *I*, where δ controls the contrast and σ controls the width, and *a* and *b* index the distance away from the center of the filter

(A5) $$I_{a,b}=\{\begin{array}{ll} -\delta e^{-\frac{a^{2}\;+\;b^{2}}{\sigma^{2}}} & \text{if }a\neq 0\text{ or }b\neq 0,\\ 1-\sum_{\begin{array}{c} a\;\neq \;0\\ b\;\neq \;0 \end{array}}I_{a,b} & \text{if }a=0\text{ and }b=0. \end{array}$$

Typical lateral inhibition parameters are given in Table A2.

**Table A2:** **Lateral inhibition parameters**.

**Layer**	**1**	**2**	**3**	**4**
Radius, σ	1.38	2.7	4.0	6.0
Contrast, δ	1.5	1.5	1.6	1.4

Next, contrast enhancement is applied by means of a sigmoid activation function

(A6) $$y=f^{sigmoid}(r)=\frac{1}{1+e^{-2\beta (r\;-\;\alpha )}}$$

where *r* is the activation (or firing rate) after lateral inhibition, *y* is the firing rate after contrast enhancement, and α and β are the sigmoid threshold and slope respectively. The parameters α and β are constant within each layer, although α is adjusted to control the sparseness of the firing rates. The sparseness *a* of the firing within a layer can be defined, by extending the binary notion of the proportion of neurons that are firing, as

(A7) $$a=\frac{{\left(\sum_{i\;=\;1}^{N}y_{i}/N\right)}^{2}}{\sum_{i\;=\;1}^{N}y_{i}^{2}/N}$$

where *y*_*i*_ is the firing rate of the *i*th neuron in the set of *N* neurons (Rolls and Treves, 1990, 1998; Rolls, 2008). For the simplified case of neurons with binarised firing rates = 0∕1, the sparseness is the proportion ∈[0, 1] of neurons that are active. For example, to set the sparseness to, say, 5%, the threshold is set to the value of the 95th percentile point of the activations within the layer. Typical parameters for the sigmoid activation function are shown in Table A3.

**Table A3:** **The sigmoid parameters used to control the global inhibition within each layer of the model**.

**Layer**	**1**	**2**	**3**	**4**
Percentile	99	98	88	95
Slope β	190	40	75	26

For these simulations we used a trace learning rule (Földiák, 1991; Rolls, 1992) to adjust the strengths of the feed-forward synaptic connections between the layers during training. The trace rule incorporates a trace ${\bar{y}}^{\tau }$ of recent neuronal activity into the postsynaptic term. The trace term reflects the recent activity of the postsynaptic cell. The effect of this is to encourage the postsynaptic cell to learn to respond to input patterns that tend to occur close together in time.

The equation of the original trace learning rule as used by Wallis and Rolls (1997) is the following

(A8) $$\Delta w_{j}=\alpha {\bar{y}}^{\tau }x_{j}^{\tau }$$

where the trace ${\bar{y}}^{\tau }$ is updated according to

(A9) $${\bar{y}}^{\tau }=(1-\eta )y^{\tau }+\eta {\bar{y}}^{\tau -1}$$

and we have the following definitions

**Table:**

*x*_*j*_:	*j*^*th*^ input to the neuron.	*y*:	Output from the neuron.
${\bar{y}}^{\tau }$:	Trace value of the output of the neuron at time step τ.	α:	Learning rate. Annealed between unity and zero.
*w*_*j*_:	Synaptic weight between *j*^*th*^ input and the neuron.	η:	Trace value. The optimal value varies with presentation sequence length.

The parameter η may be set in the interval [0, 1]. For our simulations the trace learning η is set to 0.8. If η = 0 then the Equation (A8) becomes the standard Hebb rule

(A10) $$\Delta w_{j}=\alpha y^{\tau }x_{j}^{\tau }.$$

However, the version of the trace rule used in this paper only includes the trace of activity from the immediately preceding timestep, as used in other studies (Rolls and Milward, 2000; Rolls and Stringer, 2001) for improving the performance of the standard trace rule and enhancing the effect of the invariance representation. Thus, the rule takes now the following form

(A11) $$\Delta w_{j}=\alpha {\bar{y}}^{\tau -1}x_{j}^{\tau }.$$

Neuronal mechanisms that might support trace learning in the brain have been previously discussed (Rolls, 1992; Wallis and Rolls, 1997). To restrict and limit the growth of each neuron's synaptic weight vector, **w**_*i*_ for the *i*th neuron, its length is normalized at the end of each timestep during training as is usual in competitive learning (Hertz et al., 1991). Normalization is required to ensure that the same set of neurons do not always win the competition. Neurophysiological evidence for synaptic weight normalization has been presented (Royer and Paré, 2003).

#### A.2. Information theory measures

Single and multiple cell information theoretic measures are used to assess the network's performance. Both measures help to determine whether individual cells in the output layer are able to respond to a specific target location in a hand-centered frame of reference over a number of different retinal locations. In previous VisNet studies, the single cell information measure has been applied to individual cells in the last layer of the network and measures how much information is available from the response of a single cell about which stimulus was shown. In this current study, a stimulus is defined as one of the different hand-object configurations. If an output neuron responds to just one of the spatial configurations, and the cell responds to this configuration across all tested retinal locations, then the cell will convey maximal single cell information. The amount of information carried by a single cell about a stimulus is computed using the following formula

(A12) $$I(s,R)=\sum_{r\in R}P(r|s)\log_{2}\frac{P(r|s)}{P(r)}$$

where the stimulus-specific information *I*(*s, R*) is the amount of information the set of responses *R* of a single cell has about a specific stimulus (i.e., target location with respect to the hand) *s*, while the set of responses *R* corresponds to the firing rate *y* of a cell to each of the stimuli (i.e., hand-object configurations) presented in all tested retinal locations. Further details of how the single cell information is calculated are provided in the literature (Rolls et al., 1997a; Rolls and Milward, 2000; Rolls, 2008).

The maximum single cell information measure is

(A13) $$\text{Max}.\text{ single cell info}.\;={\text{log}}_{2}(\text{Number of stimuli}).$$

For example, when we present 5 stimuli during testing, (i.e., spatial configurations of the hand and the test object), the maximum single cell information measure is 2.32 bits. When we present 6 target stimuli, the maximum single cell information measure is 2.58 bits. The cell reaches the maximal information when it responds selectively to just one of the hand-object spatial configurations, and responds to that spatial configuration across all the tested retinal positions.

On the other hand, the multiple-cell information computes the average amount of information about which stimulus was presented obtained from the responses of all the output cells. This procedure is used to verify whether, across the population of cells, there is information about all of testing stimuli (i.e., hand-object configurations) shown. Procedures for calculating the multiple cell information measure have been described in detail by Rolls et al. (1997b), Rolls and Milward (2000). In brief, from a single presentation of a stimulus, we calculate the average amount of information obtained from the responses of all the cells regarding which stimulus is shown. This is achieved through a decoding procedure that estimates which stimulus *s*′ gives rise to the particular firing rate response vector on each trial. A probability table of the real stimuli s and the decoded stimuli *s*′ is then constructed. From this probability table, the mutual information is calculated as

(A14) $$I(S,S\prime )=\sum_{s,s\prime }P(s,s\prime )\log_{2}\frac{P(s,s\prime )}{P(s)P(s\prime )}.$$

Multiple cell information values are calculated for the subset of cells which, according to the single cell analysis, have the most information about which stimulus (i.e., hand-object configuration) is shown. In particular, the multiple cell information is calculated from five cells for each stimulus that had the most single cell information about that stimulus. For example, in simulations with six target locations this results in a population of 30 cells. Previous research (Stringer and Rolls, 2000) found this to be a sufficiently large subset to demonstrate that shift invariant representations of each stimulus presented during testing were formed, and that each stimulus could be uniquely identified.

#### A.3. Data sharing

The VisNet simulator can be downloaded from https://github.com/bedeho/VisBack.
